# Evaluating the Ecotoxicological Effects of Microplastics on Terrestrial Passerines: Insights from Eurasian Tree Sparrows

**DOI:** 10.3390/toxics14050407

**Published:** 2026-05-08

**Authors:** Mo Li, Jun Wang, Weiyue Meng, Liqiang Du, Dongming Li, Yanfeng Sun

**Affiliations:** 1 College of Life Sciences, Cangzhou Normal University, Cangzhou 061001, China; 2 Hebei Key Laboratory of Bohai Rim Biomass Materials, Cangzhou Normal University, Cangzhou 061001, China; 3 Hebei Key Laboratory of Animal Physiology, Biochemistry and Molecular Biology, Hebei Collaborative Innovation Center for Eco-Environment, Ministry of Education Key Laboratory of Molecular and Cellular Biology, College of Life Sciences, Hebei Normal University, Shijiazhuang 050024, China; 4 College of Marine Resources & Environment, Hebei Normal University of Science & Technology, Qinhuangdao 066600, China; 5 Ocean College, Hebei Agricultural University, Qinhuangdao 066003, China

**Keywords:** microplastic, physiological status, gut microbiota, *Passer montanus*

## Abstract

Microplastic (MP) pollution poses a threat to wild animals, but its toxicological impact on terrestrial passerines remains unclear. To address this gap, we conducted the first systematic study investigating how microplastic particle size and dosage jointly influence gut microbiota and multi-system physiological functions in a small terrestrial bird. Eurasian tree sparrows (*Passer montanus*) were exposed to polystyrene microplastics (PS-MPs) of two particle sizes (0.5 and 15 μm in diameter) and two dosages (100 and 500 μg/d) via oral ingestion for 21 days. After exposure, body status, peripheral blood cell profiles, organ indices, intestinal histomorphology, oxidative stress, and barrier integrity markers displayed no significant changes compared with the control group. In the gut microbiota, large PS-MP particles significantly enhanced microbial species richness and phylogenetic diversity, and their effect was more pronounced than that of small ones. Additionally, structural alterations and distinct community compositions emerged across groups. Both particle size and dosage affected gut microbial composition and taxa abundance, with particle size exhibiting a relatively stronger effect. However, the relative abundance of the top 10 dominant phyla and predicted microbial functional profiles exhibited no significant intergroup differences. In summary, short-term PS-MP exposure primarily impacts the gut microbial structure of Eurasian tree sparrows without disrupting their key physiological functions. This suggests that the birds possess a certain buffering capacity against short-term PS-MP stress, though their long-term ecological tolerance to complex, real-world MP mixtures remains to be further investigated.

## 1. Introduction

Plastics have been extensively used in daily life due to their relatively low price and excellent physicochemical properties (water resistance and low density) [1]. It is estimated that the world generates approximately 400 million tons of plastic waste annually, and this amount is anticipated to rise dramatically in the future [2]. Meanwhile, large volumes of plastic waste, which are not processed effectively (limited recycling, indiscriminate disposal, and poor regulation), are released into natural ecosystems [3]. This plastic debris can be broken into smaller particles by biological processes, mechanical breakdown, and ultraviolet radiation [4], resulting in persistent deleterious impacts on the natural ecosystem. Of the diverse sizes of plastic particles, microplastics (MPs, plastic particles < 5 mm in diameter, including nanoplastics, which are <1 μm) are receiving much attention [5].

MPs may be transferred to living organisms in multiple ways [6]. For example, some lower-trophic organisms, including snails and fish, can accidentally ingest MPs and subsequently transfer them to higher-level species in the food chain [7,8,9]. MPs can also penetrate the stele of crop plants and accumulate in the edible parts, providing a potential pathway for entering the human body [10]. A growing number of studies on aquatic organisms and model mice have revealed that MP ingestion has many toxicological impacts, including growth inhibition, oxidative stress, tissue injury, disrupted gut microbiota, and reduced swimming capability in grass carp (*Ctenopharyngodon idellus*) [11,12], metabolic disorder in mice and zebrafish (*Danio rerio*) [13,14], as well as immune dysfunction in mice [15]. Nevertheless, the toxicological effects of MPs on terrestrial vertebrates, especially non-model animals, have not been adequately studied until recently [16]. Notably, compared to aquatic organisms, terrestrial species may be exposed to higher levels of MP contamination, given that MP contamination on land has been estimated to be 4–23 times higher than in water [17]. Accordingly, organisms in terrestrial environments may need to adjust the baselines of certain physiological systems to adapt to this potential pressure.

The toxicity of MPs is associated with exposure concentration, size distribution, exposure time, and chemical composition, etc. [18]. Plastic particles of smaller size can travel more easily across the intestinal mucus barrier and translocate to other tissues than larger-sized particles [5], causing tissue damage and biological dysfunction [19]. Due to differences in uptake rates, the toxicity mechanisms of MPs of different sizes also change. For example, in the marine medaka (*Oryzias melastigma*), exposure to 50 nm polystyrene nanobeads induces stronger oxidative stress than exposure to 45 μm MP, while the latter causes more severe intestinal damage and more significant alterations of the gut microbiota composition than the former [20]. Whether this size-dependent toxicity impact will also occur in terrestrial fauna remains unclear at present.

Birds are endothermic organisms widely distributed across various habitats worldwide. As the top or near-top predators of most natural ecosystems, birds are particularly susceptible to the biomagnification process of plastic particles [21,22,23]. Wilcox et al. (2015) estimated that more than 90% of seabirds carry plastic particles in their guts, and this is expected to rise to 99% of all seabird species by 2050 [22]. In addition, studies on raptors (63 deceased birds) and a comprehensive survey of terrestrial birds (63 species) across diverse habitats, behaviors, and morphologies in the United States have both found that microplastic contamination in terrestrial birds has reached a severe level of near-ubiquity [23]. Despite the high prevalence of MPs, their associated effects on bird health are still scarce [24]. Recently, several laboratory studies confirmed that exposure to MPs can result in reduced meat quality, lipid metabolism disorders, neurotransmission disturbances, and myocardial dysplasia in domestic chickens and Japanese quails (*Coturnix japonica*) [25,26,27,28]. It should be noted that some of these studies may be better described as controlled toxicological/mechanistic exposures than environmentally relevant exposure levels, especially where high dosages are included. Consequently, their findings are valuable for mechanistic hazard identification, but should not all be interpreted as directly representative of typical or likely environmental exposures.

The Eurasian tree sparrow (*Passer montanus*) is a terrestrial-dwelling bird species with widespread distribution across Eurasia [29]. As a typical human commensal species, this sparrow inhabits highly anthropized zones and is severely impacted by anthropogenic environmental pollution [30]. Prior studies on this species have demonstrated that the environmental pollution (artificial light, air, and metal pollution) can induce a series of alterations in behavioral rhythm, gut microbiota, melatonin release, egg characteristics, genetic diversity, immunological and antioxidant capacities, and hematological parameters [30,31,32,33]. Therefore, Eurasian tree sparrows can be regarded as an ideal indicator for assessing MP-induced toxicological effects in wildlife.

Polystyrene is one of the most used polymers worldwide and can serve, in particulate form, as a model for detecting the bioaccumulation and toxicity impact of MPs in organisms [34]. In this research, adult Eurasian tree sparrows were continuously fed PS-MPs for 21 days under laboratory conditions. To assess the effects of PS-MPs on the overall state of the body, peripheral blood cell composition, intestinal histomorphology, oxidative stress, barrier integrity, and gut microbiota homeostasis, multiple indicators were computed. The acquired results serve as a foundation for further investigations into the harmful impacts of MPs on terrestrial ecosystems and help give primary information on the possible threats that MPs pose to Eurasian tree sparrows.

## 2. Materials and Methods

### 2.1. PS-MPs

Two different sizes (0.5 and 15 μm in diameter) of PS-MPs were obtained from BaseLine Chromtech Research Center (http://www.qiuhuan.com (accessed on 10 September 2025), Tianjin, China) and stored at 4 °C as a stock suspension (2.5% *w*/*v*). Before use, the emulsions were freshly prepared with sterilized deionized water and ultrasonicated. The initial size and morphological characteristics of PS-MPs were verified using a scanning electron microscope (SEM, Hitachi SU8010, Hitachi High-Tech Co., Tokyo, Japan, Figure S1).

### 2.2. Experimental Protocol, Sampling, and Ethics Statements

Forty-eight free-living Eurasian tree sparrows were captured using mist nets in October 2025 in Cangzhou, Hebei province, China (37°59′47″ N, 117°5′36″ E). After capture, the birds were transferred to a laboratory and housed individually in cages at room temperature and with natural light. After 7 days of acclimation, the sparrows were randomly divided into 5 groups, including a control group (C; 0 μg/d PS-MPs; *n* = 10, 5 males and 5 females), large particle–low dosage group (LL; 15 μm, 100 μg/d PS-MPs; *n* = 9, 5 males and 4 females), large particle–high dosage group (LH; 15 μm, 500 μg/d PS-MPs; *n* = 9, 6 males and 3 females), small particle–low dosage group (SL; 0.5 μm, 100 μg/d PS-MPs; *n* = 10, 4 males and 6 females) and small particle–high dosage group (SH; 0.5 μm, 500 μg/d PS-MPs; *n* = 10, 6 males and 4 females). The particle sizes and dosages of MPs in the present study were carefully selected to ensure ecological relevance. The chosen sizes (0.5 μm and 15 μm) were based on published soil monitoring data from the North China Plain, which showed that 49% of particles are <15 μm [35]. These size fractions represent the dominant micron and nanoscale components with high biological translocation potential. The dosages used in this study (100 μg/day and 500 μg/day) were adapted from terrestrial mammal exposure protocols [36]. In the birds used here (mean body weight 17.32 g), these correspond to approximately 5.77 and 28.87 mg/kg bw/day, respectively. Therefore, these treatments should not be described as typical environmentally realistic intakes. Instead, the 100 μg/day dose is better interpreted as a high-end/worst-case environmental exposure, while the 500 μg/day dose represents a high toxicological challenge dose relevant for hazard identification under extreme contamination scenarios.

PS-MPs were administered to the birds orally once daily for 21 days (feeding volume: 20 μL), while those of the control group received equivalent volumes of sterilized deionized water. The 21-day exposure duration was also selected based on the microplastic toxicity test paradigm in terrestrial vertebrates [36]. During the acclimation and PS-MP treatment period, body mass, core temperature, and food intake of each bird were monitored daily. Body mass and food intake were measured using a portable electronic balance (to the nearest 0.1 g), with food intake calculated as the difference between total feed provided and leftovers on the next day. Core temperature was recorded to the nearest 0.1 °C by inserting a 30-gauge type K thermocouple attached to a thermometer into the cloaca to a depth of about 10 mm. All birds received commercial feed and water *ad libitum* throughout the experiments. All treatments and measurements were performed between 8:00 and 10:00 am.

Following the 21-day exposure to PS-MPs, the alar vein of each bird was punctured with a 26-gauge needle to obtain about 10 μL of blood, which was then used for blood smears for subsequent blood cell count analysis. Afterwards, the birds were euthanized using phenobarbitone (7.5 μL/g body weight), and their hearts, livers, gizzards, and intestines were dissected. The gizzard was incised, rinsed with ice-cold saline to remove food residues, and then weighed. Intestine contents were collected with sterile cotton swabs for genomic DNA extraction and 16S rRNA sequencing. After the collection of contents, the intestines were also rinsed with saline, weighed, and divided into two portions. One aliquot was quickly frozen in liquid nitrogen and then stored at −80 °C for use in further analyses. The same procedure was followed for the liver, gizzard, and intestinal contents. The other aliquot was preserved in 4% (*w*/*v*) precooled paraformaldehyde solution for subsequent histological examination. All organs were blotted dry and weighed to the nearest 0.0001 g using an analytical balance. The organ index was calculated as the ratio of organ weight and body mass.

The Forestry Administration of Hebei Province permitted the capture of the birds, and all experimental procedures received approval from the Ethics Committee for Experimental Animals, Hebei Agricultural University, China (approval number: 2025078).

### 2.3. Biochemical Analysis

To assess oxidative stress and the barrier integrity of the digestive system, levels of malondialdehyde (MDA) in the liver, intestine, and gizzard, as well as catalase (CAT) and diamine oxidase (DAO) in the liver and intestine, were measured. Tissues were weighed and fully homogenized with ice-cold saline (1:9 *w*/*v*) using a glass homogenizer. The homogenates were then centrifuged at 3000× *g* for 15 min at 4 °C. The resulting supernatants were aliquoted and stored at 4 °C until required for biochemical assays. All measurements were performed using a microplate reader (Multiskan SkyHigh, Thermo Fisher Scientific Inc., Waltham, MA, USA) with commercially available kits as directed by the manufacturer (Cat. Nos: CAT, A007-1-1; MDA, A003-1-1; DAO, A088-3-1) at the Bioengineering Institute, Nanjing, China. The protein concentration of the supernatants was tested using the BCA protein assay kit (E112-01, Vazyme Biotech Corporation, Nanjing, China). Each parameter was measured in duplicate.

### 2.4. Histological Analysis of Intestines

After being preserved in 4% (*w*/*v*) paraformaldehyde solution for 24 h, the intestine samples were dehydrated using a graded series of ethanol solutions (50–100%) and cleared with xylene, embedded in paraffin wax at 45 °C, and sectioned at 4 μm thickness using a rotation microtome (MICROM HM330, MICROM International GmbH, Walldorf, Germany). Each sample section was further stained with Hematoxylin and Eosin (H&E), embedded in resin, and examined under a light microscope (Olympus BX51, Olympus Corporation, Tokyo, Japan) equipped with a camera (Olympus DP20, Olympus, Tokyo, Japan). Histological indices, including outer and inner circumference, number of villi per section, villus height and width, villus absorptive surface area, mucosa thickness, and muscularis thickness, were measured using ImageJ software (version 1.52), as previously described [37]. Histological lesions, including leucocyte infiltration, crypt or villus cell loss, cryptitis, hyperemia, denudation of villi, and fusion, were also observed, as previously described [38,39,40]. The treatment groups were not disclosed to the observers performing the analyses.

### 2.5. Blood Cell Count and Classification

Blood smears were air-dried, stained with Wright’s stain, mounted with neutral resin, and photographed with a light microscope under oil immersion at a 1000× magnification. The number of different erythrocytes (including normal erythrocytes, immature erythrocytes, polychromatic erythrocytes, hypochromic erythrocytes, microcytes, and abnormally shaped erythrocytes), leukocytes (including heterophils, eosinophils, basophils, monocytes, and lymphocytes), and thrombocytes was recorded [30]. Next, the proportions of erythrocytes, leukocytes, and thrombocytes in total hemocytes (sum of the erythrocytes, leukocytes, and thrombocytes) were calculated. Additionally, we computed the proportions of erythrocyte subtypes within the total erythrocyte population, leukocyte subpopulations within the total leukocyte count, and the heterophil-to-lymphocyte ratio (H/L). To obtain robust results, we observed about 18,241 blood cells (10,828–33,974) and an additional 92 (0–161) leukocytes (only used for calculating proportional distributions of leukocyte subtypes). The visual fields of the blood smear were randomly selected, and observers were blinded to the treatment groups.

### 2.6. DNA Extraction, 16S rRNA Gene Amplicon Sequencing and Bioinformatic Analysis

The experimental protocols (DNA extraction, 16S rRNA gene amplification, and sequencing) and the bioinformatics analysis (sequence processing, quality control, OTU clustering, and taxonomic annotation) were conducted as described before [41]. Briefly, total genomic DNA was extracted from intestinal contents, and the V4 region of the 16S rRNA gene was amplified. Separated and purified PCR products were sequenced on an Illumina HiSeq 2500 platform. Following merging and quality filtering, the raw reads were clustered into OTUs at 97% sequence similarity and taxonomically classified using the Greengenes database. The OTU abundances were rarefied to the minimum sequencing depth, and rarefaction curves were generated.

### 2.7. Statistical Analysis

To investigate variations in the gut microbiota among groups, α-diversity (Observed_species, Chao1, ACE, Shannon, Simpson, and PD_whole_tree indices) and β-diversity (binary Jaccard distance) indices were calculated. Permutational multivariate analysis of variance (PERMANOVA) was used for statistical comparison (999 permutations), and principal coordinate analysis (PCoA) for visualization. Linear discriminant analysis (LDA) effect size (LEfSe) was conducted to discover significantly different micro-populations among groups (LDA score > 3.0, *adjusted p* < 0.05). Canonical correspondence analysis (CCA) was performed using Canoco 5 to elucidate the impact of the MPs on microbial communities. The functional profiles of microbial communities were predicted using PICRUSt2, with the Kyoto Encyclopedia of Genes and Genomes (KEGG) database used for annotation. All the above analyses were carried out in R (version 4.4.1, https://www.R-project.org (accessed on 2 August 2024)) except for CCA and functional profile prediction.

The changes in body mass, core temperature, and food intake of birds during the experiments were statistically evaluated using repeated-measures ANOVA, with time as a within-subject repeated factor. Organ indices, intestinal histological indices, peripheral blood cell profiles, and biochemical parameters in the liver, intestine, and gizzard were compared using two-way ANOVA. Group, sex, and the interaction between group and sex were specified as between-subject fixed effects. Comparisons of the top 10 phyla and α-diversity among the different groups were conducted using one-way ANOVA or the Kruskal–Wallis rank test. Tests for homogeneity of variance and normality of the data were performed before comparative analysis. Statistical analyses were conducted using the Statistical Package for the Social Sciences software (SPSS, version 23.0, SPSS Inc., Chicago, IL, USA). Bonferroni post hoc tests were employed for multiple comparisons. *p*-values were adjusted using the Benjamini–Hochberg (BH) method to control the false discovery rate in R, and the threshold for statistical significance was established at 0.05. Figures were generated using R and GraphPad Prism (version 8.0) software.

## 3. Results

### 3.1. Changes in Body Mass, Core Temperature, Food Intake, and Hematological Characteristics

**Figure 1:**
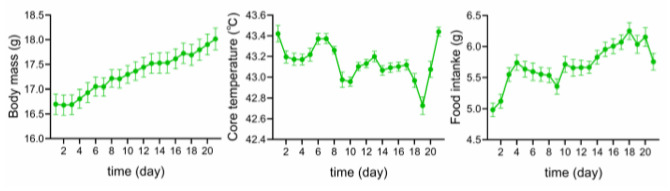
Temporal variation in body mass, core temperature, and food intake in Eurasian tree sparrows (*Passer montanus*) over the 21-day experimental period. Data are presented as mean ± SEM.

Body mass, core temperature, and food intake of the sparrows showed significant temporal variations. However, no significant effects of group, sex, or the interaction between group and sex were detected (Table 1). Pairwise comparisons revealed a significant increasing trend in body mass over time. Core temperature and food intake displayed temporal fluctuations throughout the experiments, and food intake demonstrated an overall increasing trend (Table S1, Figure 1).

In peripheral blood, the effects of group, sex, and the interaction between group and sex on the proportions of erythrocytes, leukocytes, and thrombocytes were not significant. Concurrently, these factors did not exhibit significant effects on the proportions of erythrocyte and leukocyte subtypes or the H/L ratio (Table 2).

### 3.2. Histological Response and Oxidative Stress

Group, sex, and their interaction exerted no significant effect on organ indices (heart, liver, intestine, and gizzard), MDA (liver, intestine, and gizzard), CAT, and DAO levels (liver and intestine) (Table 3). Outer and inner circumference, number of villi per section, villus height and width, villus absorptive surface area, mucosa thickness, and muscularis thickness in the intestine also presented no significant main effects of group, sex, and the interaction between group and sex (Table 3). In addition, after exposure to PS-MPs, the intestines did not display dramatic histological lesions (Figure S2).

### 3.3. Changes in Gut Microbiota Composition After PS-MPs Exposure

A total of 7,072,300 effective reads were obtained after splicing and filtering the sequencing data (Table S2), with 101,153–158,107 effective reads per sample. The number of OTUs among all samples ranged from 553 to 1074 (Table S3). After filtering data, 78,295 sequences per sample were used for subsequent analysis. The rarefaction curve confirmed that the sequencing depth was sufficient for all samples (Figure S3). Since no significant sex differences were detected across groups, the data of males and females were pooled together for subsequent analysis (Figure S4).

**Figure 2:**
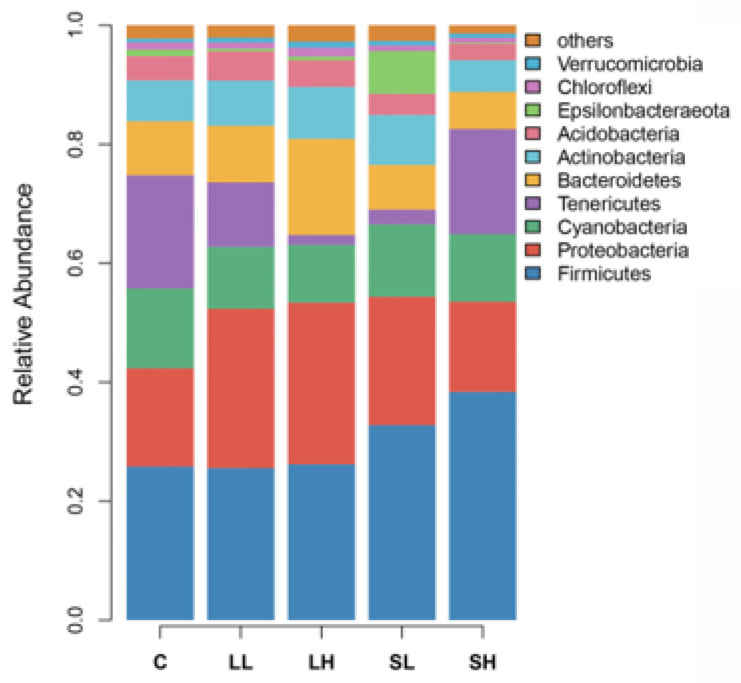
Relative abundance of the top 10 dominant phyla in the gut microbiota of Eurasian tree sparrows (*Passer montanus*) across different groups. Abbreviations: C, control group; LL, large particle–low dosage group; LH, large particle–high dosage group; SL, small particle–low dosage group; SH, small particle–high dosage group.

The gut microbiota was primarily composed of 10 phyla (Firmicutes, Proteobacteria, Cyanobacteria, Tenericutes, Bacteroidetes, Actinobacteria, Acidobacteria, Epsilonbacteraeota, Chloroflexi, and Verrucomicrobia), accounting for over 97% of the total sequence across all samples (Figure 2). None of the phyla differed significantly among groups (Table S4).

**Figure 3:**
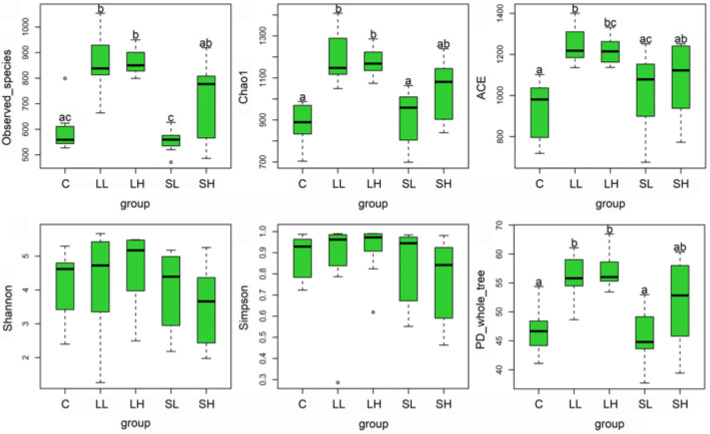
Intergroup comparisons of gut microbial α-diversity indices. The upper and lower boundaries of the box represent the third and the first quartile, respectively. The horizontal line inside the box indicates the median. The whiskers extend to the extreme values within 1.5 times the interquartile range, and the scattered dots represent discrete observations outside the main distribution. Pairwise comparisons were conducted by the Bonferroni method. Different lowercase letters above the boxes indicate significant differences among groups after BH correction. Abbreviations: C, control group; LL, large particle–low dosage group; LH, large particle–high dosage group; SL, small particle–low dosage group; SH, small particle–high dosage group.

α-diversity indices, including Observed_species, Chao1, ACE, and PD_whole_tree, displayed substantial intergroup differences, while the Shannon and Simpson indices did not (Table 4, Figure 3). Compared to the C group, the LL and LH groups had higher Observed_species, Chao1, ACE, and PD_whole_tree. SL and SH groups did not demonstrate any significant change compared with the C group. Pairwise comparisons of the α-diversity indices between LL and LH groups, SL and SH groups, and LH and SH groups also displayed no significant differences, except for a higher Observed_species in the SH group than the SL group. The LL group exhibited higher Observed_species, Chao1, ACE, and PD_whole_tree than the SL group (Table 4, Figure 3).

**Figure 4:**
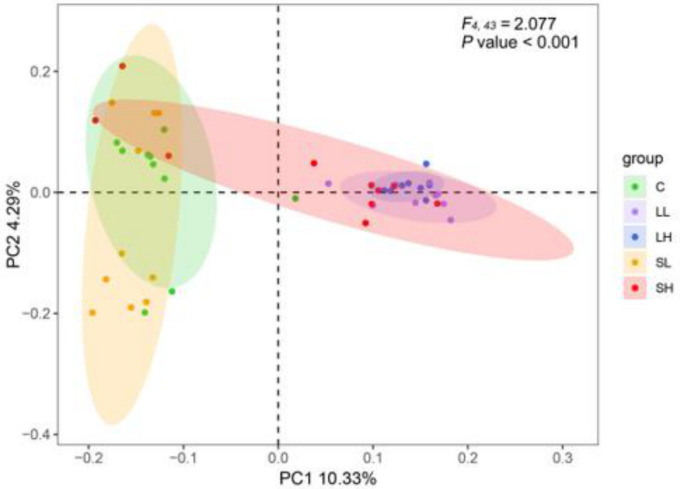
Gut microbiota β-diversity patterns visualized through principal coordinate analysis (PCoA) based on Binary-Jaccard distances. Each point represents an individual sample. The statistical differences between groups were determined by PERMANOVA. Abbreviations: C, control group; LL, large particle–low dosage group; LH, large particle–high dosage group; SL, small particle–low dosage group; SH, small particle–high dosage group.

**Figure 5:**
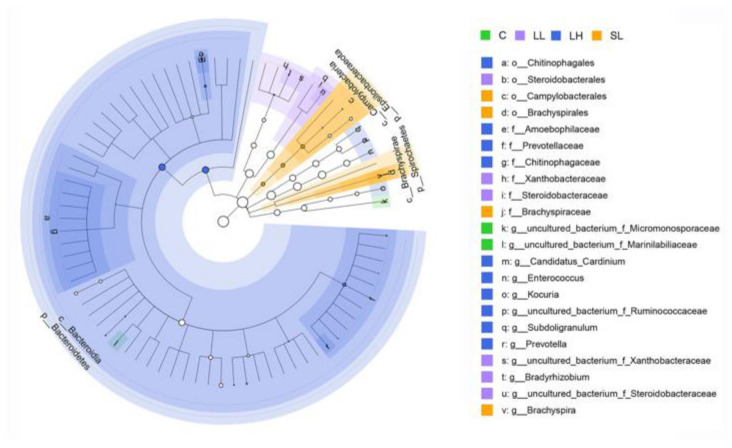
The circular cladogram highlighting differential bacterial taxa selected by linear discriminant analysis effect size (LEfSe) analysis (LDA > 3.0, *adjusted p* < 0.05). Abbreviations: C, control group; LL, large particle–low dosage group; LH, large particle–high dosage group; SL, small particle–low dosage group.

β-diversity analysis revealed significant intragroup variation in gut microbial composition (*F*_4,43_ = 2.077, *p* < 0.001; Figure 4, Table S5). LEfSe analysis further identified the biomarkers across various taxonomic levels among the different groups. The C group conserved a significantly higher relative abundance of *uncultured_bacterium_f_Micromonosporaceae* and *uncultured_bacterium_f_Marinilabiliaceae* at the genus level. The LL group exhibited a higher abundance of Steroidobacterales at the order level, Xanthobacteraceae and Steroidobacteraceae at the family level, *Bradyrhizobium*, *uncultured_bacterium_f_Xanthobacteraceae*, and *uncultured_bacterium_f_Steroidobacteraceae* at the genus level. The LH group displayed the most abundant biomarkers relative to other groups, exhibiting higher abundance of Bacteroidetes at the phylum level, Bacteroidia at the class level, Chitinophagales at the order level, Amoebophilaceae, Prevotellaceae, and Chitinophagaceae at the family level, and *Candidatus_Cardinium*, *Enterococcus*, *Kocuria*, *uncultured_bacterium_f_Ruminococcaceae*, *Subdoligranulum*, and *Prevotella* at the genus level. The SL group exhibited higher levels of Epsilonbacteraeota, and Spirochaetes at the phylum level, Campylobacteria, and Brachyspirae at the class level, Campylobacterales, and Brachyspirales at the order level, Brachyspiraceae at the family level, and *Brachyspira* at the genus level (Figure 5, Table S6). However, the predicted function profiles displayed no significant difference across experimental groups (Table S7, Figure S5).

**Figure 6:**
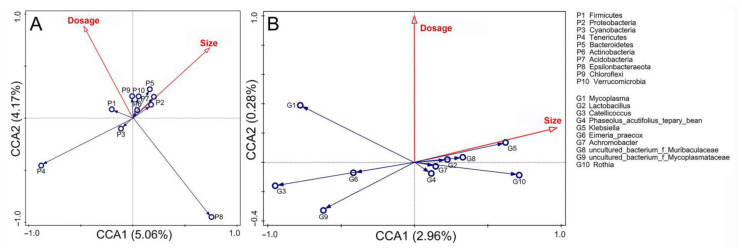
Canonical correspondence analysis (CCA) showing relationships between the PS-MP properties (particle size and dosage) and the top 10 dominant microbial phyla (**A**), genus (**B**). Red arrows represent the PS-MP properties, blue circles and arrows represent dominant microbial taxa.

CCA revealed that the abundance of microbial taxa was significantly influenced by PS-MPs dosage and size at the phylum level (*F* = 2.3, *p* = 0.046). Furthermore, PS-MP size tended to exhibit relatively stronger correlations with microbial taxa abundance than dosage did. Specifically, the abundance of Epsilonbacteraeota and Tenericutes was negatively correlated with PS-MP dosage and size, respectively. However, most other dominant phyla tended to show a closer association with PS-MP size than with dosage, for instance, Proteobacteria, Bacteroidetes, and Acidobacteria (Figure 6A). Although the model was not statistically significant at the genus level (*F* = 0.8, *p* = 0.686), the abundances of dominant genera also appear to be more closely related to PS-MP size than to dosage, as exemplified by *Catellicoccus* and *Klebsiella* (Figure 6B).

## 4. Discussion

### 4.1. Physiological Stability After PS-MPs Exposure

Body mass, core temperature, food intake, and hemogram are common indices indicating physiological conditions of animals [30,42]. In mammalian studies, MP exposure can result in weight loss, decreased appetite, and hematopoietic system damage [43,44]. Additionally, physiological alterations induced by exposure to MPs, such as inflammatory responses and metabolic disorders, may also disturb thermoregulation, leading to changes in body temperature [45,46]. However, our findings indicated that body mass, core temperature, food intake, and hematological parameters of the sparrows remained stable following ingestion of the PS-MPs. Similar results in final body mass were also observed in Japanese quail, which experienced oral exposure to MPs of two distinct size classes (3 mm and <125 μm) at a total dose of 600 mg per bird over 5 weeks [47]. These results suggest that (1) the physiological impacts of MPs are not universally severe across different animal taxa; (2) small terrestrial birds exhibit limited physiological disruption under short-term PS-MP exposure, potentially due to their ability to excrete MP particles and mitigate toxic effects.

The digestive tract and the liver are important target organs for MPs [36]. Ingested MPs can also cross the mucus layer of the intestine via endocytosis, transcytosis, and paracellular transport, translocate into the bloodstream, and penetrate other organs, causing structural damage, functional disorders, and oxidative stress [12,48,49]. Here, exposure to PS-MPs did not cause any detectable changes in organ indices, intestinal histological morphology, barrier integrity, and oxidative stress markers of the digestive system. This can be explained by the following main factors. First, as a small passerine bird, the Eurasian tree sparrow possesses a relatively short digestive tract and exhibits a high food passage rate to support its high metabolic demands [50]. This rapid passage may shorten the retention time of PS-MPs and reduce their toxic effects. As PS-MPs might be excreted before they can accumulate to a concentration sufficient to cause tissue damage or functional abnormalities, the rapid passage may also decrease the likelihood of PS-MPs interacting with intestinal epithelial cells or entering the circulatory system to impact other organs. Second, many bird species, including the Eurasian tree sparrows, typically ingest grit to aid food digestion or provide supplemental nutrients [51]. They have evolved a suite of adaptive physiological traits to cope with ingested exogenous particles in the digestive tract, for instance, a mucus layer of the intestinal mucosa and a muscular gizzard with a well-developed cuticle lining, which can efficiently grind and fragment ingested materials, thereby minimizing physical irritation and accumulation of particles in the intestine [52,53]. These characteristics may provide a certain degree of resistance and repair capability against MPs.

### 4.2. Gut Microbiota Alterations

We did not observe significant intergroup differences in the relative abundance of the top 10 dominant phyla, suggesting that PS-MP exposure did not disrupt the core structure of the microbiota at the phylum level. Compared with the C group, LL and LH groups had higher Observed_species, Chao1, ACE, and PD_whole_tree, while the SL and SH groups showed no significant differences from the C group. In addition, though LH and SH groups did not exhibit a significant difference in α-diversity, the LL group displayed significantly higher Observed_species, Chao1, ACE, and PD_whole_tree than the SL group. These results suggest that large PS-MP particles tend to enhance microbial species richness and phylogenetic diversity, and their effect is more pronounced than that of small ones. Regarding the dosage effects, only the SH group showed higher Observed_species than the SL group, while comparisons between LL and LH, SL and SH displayed no other differences. These results indicate that exposure to higher PS-MP dosage can promote microbial species richness, but the dosage effect of PS-MPs is relatively mild compared to the size effect. The discrepancy between PS-MP dosage and size effects likely arises from their distinct mechanisms of action. Particle size might influence their intestinal absorption, distribution, retention time, interactions with mucus and epithelium, and their surface adsorption and carrier capacity, thereby exerting varying effects on the gut microbiota [54,55,56]. In contrast, dosage may act via a more restricted mechanism related to chemical stress thresholds [57].

β-diversity analysis further confirmed that exposure to PS-MPs significantly changed the overall community structure of the gut microbiota, with significant differences in community composition among all groups. LEfSe analysis identified microbial biomarkers associated with MPs ingestion. Xanthobacteraceae and *Bradyrhizobium* are involved in nitrogen fixation [58,59]. Steroidobacteraceae, a family belonging to the order Steroidobacterales, is likely associated with steroid degradation and denitrification processes [60,61]. Accordingly, the increased abundance of these microbial taxa in the LL group might drive alterations in lipid and nitrogen metabolism. The LH group exhibited the most abundant biomarkers, among which Bacteroidetes, Bacteroidia, Prevotellaceae, Chitinophagales, *Enterococcus*, and *Subdoligranulum* may produce a range of secondary metabolites, such as short-chain fatty acids, succinate, N-Acetylglucosamine (GlcNAc), antimicrobial, and antifungal compounds [62,63,64,65,66]. In addition, *Kocuria* and *Prevotella* might also participate in the breakdown of lipids and proteins [67,68]. Given that these resulting secondary metabolites, for instance, short-chain fatty acids, can supply energy and metabolic substrates, regulate the immune status and intestinal barrier function of the host [69], changes in the abundance of these microbial taxa might induce potential alterations in the energy balance and physiological homeostasis of Eurasian tree sparrows. The SL group was characterized by a higher abundance of Epsilonbacteraeota, Campylobacteria, Campylobacterales, Spirochaetes, Brachyspirae, Brachyspirales, Brachyspiraceae, and *Brachyspira*. Campylobacterales, an order classified under the class Campylobacteria and phylum Epsilonbacteraeota, is commonly found in the intestinal tract of a wide variety of domestic and wild animals [70]. This order encompasses *Campylobacter*, which is recognized as a pathogen responsible for diarrheal diseases [71]. *Brachyspira*, belonging to the family Brachyspiraceae, order Spirochaetales, and phylum Spirochaetes, also conserves many various causative agents of intestinal spirochaetosis and swine dysentery [72]. This suggests that the SL group may have distinct gut microbiota characteristics associated with potential pathogenic risks or specific physiological states linked to these bacteria.

Unexpectedly, despite composition changes after PS-MP exposure, we observed no dramatic alterations in the predicted functional profiles of the gut microbiota. This discrepancy may be attributed to functional redundancy within avian gut microbial communities [73]. The complementary metabolic capabilities among different microbial taxa may buffer the disruptive effects of PS-MPs, enabling the gut microbiota to retain functional stability. Similar patterns have also been observed in previous avian microbiome studies. For instance, a metagenomic study on Scaly-sided Merganser (*Mergus squamatus*) revealed significant compositional divergence in gut microbiota among populations inhabiting different rivers, but the functional characteristics remained conserved [74]. Thus, the observed significant shifts in gut microbiota composition likely represent a sensitive yet functionally compensated response, rather than overt toxic damage, given the stability of physiological status and gut microbiota functional profiles.

CCA revealed that the dosage and size of PS-MP played a role in shaping the abundance of microbial taxa. Epsilonbacteraeota and Tenericutes showed negative correlations with PS-MP dosage and size, respectively, suggesting that these taxa might be sensitive targets to PS-MPs. Furthermore, the correlation between microbial abundance and PS-MP size was generally stronger than that with PS-MP dosage, which aligns with the pattern observed in the α-diversity analysis. Given that MP size and dosage were set according to environmentally relevant exposure gradients, the stronger effect of particle size relative to dosage might carries ecological implications. MP size may be a more critical predictor of ecological risk for wild passerines in their natural habitats than environmental concentration. Nevertheless, the dynamic mechanisms underlying the interactions between PS-MP properties and microbial community shifts warrant further investigation.

## 5. Limitations

While our study provides valuable insights into the effects of PS-MPs on a wild passerine species, several limitations should be noted to appropriately contextualize the findings. Firstly, the use of wild-caught sparrows improved ecological relevance but introduced individual variability, and the small sample size per group may have limited the statistical power. Although all birds were captured at the same location and season, and biological variability was mitigated by randomization and acclimation, the variability remained an unavoidable source of background noise. Secondly, we did not directly quantify the uptake, retention, or tissue accumulation of PS-MPs, which restricts the precise interpretation of exposure-response relationships. Future studies combining MP tracking or quantification will help strengthen mechanistic conclusions. Finally, our study was limited to single-component (polystyrene), short-term laboratory exposure. In nature, MPs exist as complex mixtures (with additives) and co-occur with other pollutants (e.g., heavy metals, persistent organic pollutants). Long-term and multi-pathway exposure in such scenarios will likely amplify ecological risks that MPs pose to wild birds beyond what we observed in this study, underscoring the necessity for further targeted research.

## 6. Conclusions

Using the Eurasian tree sparrow as a study model, we systematically assessed the impacts of PS-MPs on physiology and gut microbiota under different exposure conditions. Specifically, the body status, organ function, intestinal structure, oxidative balance, and blood cell profiles all remained stable after 21-day PS-MP ingestion, indicating short-term PS-MP exposure did not disrupt core physiological homeostasis. However, in terms of gut microbiota, PS-MPs induced selective structural shifts, with PS-MP size exerting a relatively stronger effect than dosage, but the microbial functional profiles remained unchanged. Our findings indicate that short-term MP exposure has a limited impact on free-living terrestrial passerines, bridging a key gap in MP ecotoxicity research of birds.

## Figures and Tables

**Table 1:** Effect of time, group, sex, and interaction of group and sex on body mass, core temperature, and food intake in Eurasian tree sparrows (*Passer montanus*). *Adjusted p* represents *p*-values corrected by the BH method. *P* and *adjusted p* values < 0.05 were marked in bold. Factors were considered significant at *adjusted p* < 0.05.

Variable	Factor	*df*	*F*	*p*	*Adjusted p*
body mass	time	1.706, 64.818	45.515	**<0.001**	**<0.001**
	group	4, 38	0.114	0.977	0.978
	sex	1, 38	3.342	0.075	0.150
	group × sex	4, 38	0.111	0.978	0.978
core temperature	time	6.128, 232.851	15.741	**<0.001**	**<0.001**
	group	4, 38	0.157	0.959	0.978
	sex	1, 38	0.161	0.690	0.978
	group × sex	4, 38	0.326	0.859	0.978
food intake	time	5.516, 209.617	20.977	**<0.001**	**<0.001**
	group	4, 38	2.910	**0.034**	0.102
	sex	1, 38	2.652	0.112	0.192
	group × sex	4,38	2.517	0.057	0.137

**Table 2:** The impact of group, sex, and their interaction on peripheral blood cell composition in Eurasian tree sparrows (*Passer montanus*). *Adjusted p* represents *p*-values corrected by the BH method. Factors were considered significant at *adjusted p* < 0.05. Abbreviations: H/L, heterophil-to-lymphocyte ratio.

Variable	Factor	*df*	*F*	*p*	*Adjusted p*	Variable	Factor	*df*	*F*	*p*	*Adjusted p*
erythrocyte	group	4, 38	0.817	0.522	0.886	microcyte	group	4, 38	0.409	0.801	0.990
	sex	1, 38	0.002	0.967	0.990		sex	1, 38	0.047	0.830	0.990
	group × sex	4, 38	0.170	0.953	0.990		group × sex	4, 38	0.703	0.595	0.895
leukocyte	group	4, 38	0.315	0.866	0.990	heterophil	group	4, 38	0.925	0.460	0.869
	sex	1, 38	0.056	0.815	0.990		sex	1, 38	0.116	0.735	0.983
	group × sex	4, 38	1.249	0.307	0.869		group × sex	4, 38	0.991	0.424	0.869
thrombocyte	group	4, 38	1.166	0.341	0.869	eosinophil	group	4, 38	1.125	0.359	0.869
	sex	1, 38	0.060	0.809	0.990		sex	1, 38	0.515	0.477	0.875
	group × sex	4, 38	2.183	0.089	0.869		group × sex	4, 38	0.101	0.981	0.990
immature erythrocyte	group	4, 38	0.952	0.445	0.869	basophil	group	4, 38	0.919	0.463	0.869
	sex	1, 38	0.028	0.867	0.990		sex	1, 38	1.165	0.287	0.869
	group × sex	4, 38	2.237	0.083	0.869		group × sex	4, 38	1.031	0.404	0.869
polychromatic erythrocyte	group	4, 38	0.269	0.896	0.990	monocyte	group	4, 38	2.181	0.090	0.869
	sex	1, 38	0.085	0.772	0.990		sex	1, 38	1.165	0.287	0.869
	group × sex	4, 38	1.183	0.334	0.869		group × sex	4, 38	1.906	0.129	0.869
hypochromic erythrocyte	group	4, 38	2.185	0.089	0.869	lymphocyte	group	4, 38	1.019	0.410	0.869
	sex	1, 38	0.922	0.343	0.869		sex	1, 38	0.091	0.764	0.990
	group × sex	4, 38	0.916	0.464	0.869		group × sex	4, 38	1.393	0.255	0.869
abnormally shaped erythrocyte	group	4, 38	0.698	0.598	0.895	H/L	group	4, 38	0.698	0.598	0.895
	sex	1, 38	0.602	0.443	0.869		sex	1, 38	0.152	0.698	0.965
	group × sex	4, 38	1.334	0.275	0.869		group × sex	4, 38	1.597	0.195	0.869

**Table 3:** The impact of group, sex, and their interaction on organ index, biochemical parameters in liver, intestine, and gizzard, as well as on intestinal histomorphological indices in Eurasian tree sparrows (*Passer montanus*). *Adjusted p-*values represent *p*-values corrected by the BH method. Factors were considered significant at *adjusted p* < 0.05. Abbreviations: MDA, malondialdehyde; CAT, catalase; DAO, diamine oxidase.

Organ	Variable	Factor	*df*	*F*	*p*	*Adjusted p*	Tissue	Variable	Factor	*df*	*F*	*p*	*Adjusted p*
heart	organ index	group	4, 38	1.622	0.189	0.869		MDA	group	4, 38	1.001	0.419	0.869
		sex	1, 38	0.139	0.711	0.965			sex	1, 38	0.139	0.712	0.965
		group × sex	4, 38	0.693	0.601	0.895			group × sex	4, 38	0.282	0.888	0.990
liver	organ index	group	4, 38	0.158	0.958	0.990	intestine	outer circumference	group	4, 35	1.215	0.322	0.869
		sex	1, 38	0.742	0.395	0.869			sex	1, 35	0.215	0.646	0.940
		group × sex	4, 38	1.808	0.147	0.869			group × sex	4, 35	0.140	0.966	0.990
	MDA	group	4, 38	0.941	0.451	0.869		inner circumference	group	4, 35	1.419	0.248	0.869
		sex	1, 38	0.546	0.465	0.869			sex	1, 35	0.465	0.500	0.876
		group × sex	4, 38	0.560	0.693	0.965			group × sex	4, 35	0.581	0.679	0.965
	CAT	group	4, 38	0.706	0.593	0.895		number of villi per section	group	4, 35	0.111	0.978	0.990
		sex	1, 38	0.292	0.592	0.895			sex	1, 35	1.297	0.262	0.869
		group × sex	4, 38	1.953	0.122	0.869			group × sex	4, 35	0.420	0.793	0.990
	DAO	group	4, 38	1.815	0.146	0.869		villus height	group	4, 35	0.809	0.528	0.886
		sex	1, 38	0.030	0.863	0.990			sex	1, 35	0.050	0.824	0.990
		group × sex	4, 38	0.208	0.932	0.990			group × sex	4, 35	1.275	0.298	0.869
intestine	organ index	group	4, 38	0.687	0.606	0.895		villus width	group	4, 35	0.848	0.505	0.876
		sex	1, 38	0.037	0.849	0.990			sex	1, 35	0.897	0.350	0.869
		group × sex	4, 38	1.862	0.137	0.869			group × sex	4, 35	0.167	0.954	0.990
	MDA	group	4, 38	0.996	0.421	0.869		villus absorptive surface area	group	4, 35	1.297	0.290	0.869
		sex	1, 38	0.342	0.562	0.895			sex	1, 35	1.076	0.307	0.869
		group × sex	4, 38	1.752	0.159	0.869			group × sex	4, 35	1.106	0.369	0.869
	CAT	group	4, 38	0.943	0.450	0.869		mucosa thickness	group	4, 35	0.856	0.500	0.876
		sex	1, 38	1.265	0.268	0.869			sex	1, 35	0.007	0.936	0.990
		group × sex	4, 38	0.931	0.456	0.869			group × sex	4, 35	1.476	0.231	0.869
	DAO	group	4, 38	1.103	0.369	0.869		muscularis thickness	group	4, 35	1.166	0.342	0.869
		sex	1, 38	0.921	0.343	0.869			sex	1, 35	0.814	0.373	0.869
		group × sex	4, 38	1.896	0.131	0.869			group × sex	4, 35	1.379	0.261	0.869
gizzard	organ index	group	4, 38	1.416	0.247	0.869							
		sex	1, 38	0.0002	0.990	0.990							
		group × sex	4, 38	0.279	0.890	0.990							

**Table 4:** Effects of PS-MPs on α-diversity of gut microbiota. Data are presented as mean ± SEM. *Adjusted p* represents *p*-values corrected by the BH method. Pairwise comparisons were conducted by the Bonferroni method and corrected using the BH method. Statistical significance was defined as *adjusted p* < 0.05. *P* and *adjusted p* values < 0.05 were marked in bold. Different lowercase letters following the numbers represent significant differences among groups. Abbreviations: C, control group; LL, large particle–low dosage group; LH, large particle–high dosage group; SL, small particle–low dosage group; SH, small particle–high dosage group.

Index	C	LL	LH	SL	SH	*df*	*F/H*	*p*	*Adjusted p*
Observed_species	591.6 ± 25.468ac	858.778 ± 38.414b	859.778 ± 17.306b	556.4 ± 13.564c	719.9 ± 46.487ab	4	29.311	**<0.001**	**<0.001**
Chao1	880.45 ± 30.117a	1194.214 ± 39.958b	1183.629 ± 23.488b	919.907 ± 37.751a	1045.64 ± 44.337ab	4, 43	15.857	**<0.001**	**<0.001**
ACE	938.575 ± 44.624a	1249.935 ± 29.787b	1217.801 ± 21.425bc	1031.295 ± 55.005ac	1071.716 ± 52.212ab	4	24.989	**<0.001**	**<0.001**
Shannon	4.173 ± 0.312	4.341 ± 0.489	4.658 ± 0.351	3.959 ± 0.369	3.429 ± 0.351	4	7.813	0.099	0.115
Simpson	0.891 ± 0.031	0.864 ± 0.076	0.913 ± 0.041	0.839 ± 0.055	0.77 ± 0.06	4	7.218	0.125	0.125
PD_whole_tree	46.849 ± 1.159a	55.925 ± 1.375b	57.392 ± 1.052b	45.783 ± 1.379a	51.678 ± 2.118ab	4, 43	12.151	**<0.001**	**<0.001**

## Data Availability

The original contributions presented in this study are included in the article/Supplementary Material. Further inquiries can be directed to the corresponding authors.

## Supplementary Materials

The following supporting information can be downloaded at: https://www.mdpi.com/article/10.3390/toxics14050407/s1, Table S1: The post hoc multiple comparison for body mass, core temperature and food intake; Table S2: Reads per sample by 16S rRNA sequencing; Table S3: The OTU counts of each sample; Table S4: Relative abundance variations in the top 10 phyla; Table S5: Pairwise PERMANOVA comparisons of gut microbial β-diversity; Table S6: Differential bacterial taxa identified by Lefse analysis; Table S7: The relative frequency percentage of top 10 predicted function and pairwise comparison results; Figure S1: Scanning electron microscope images of PS-MPs; Figure S2: Intestinal tissue section; Figure S3: Rarefaction curve; Figure S4: PERMANOVA analysis between different sexes; Figure S5: Relative abundance of predicted function of microbial communities.
