# Supplementary material for: Evaluating the Ecotoxicological Effects of Microplastics on Terrestrial Passerines: Insights from Eurasian Tree Sparrows
Source: Toxics. 2026 May 8;14(5):407. doi: 10.3390/toxics14050407 (PMC13211408; doi:10.3390/toxics14050407)
Supplement: Supplementary file 1 [file toxics-14-00407-s001.zip › toxics-4226914-supplementary.pdf]

Supplementary Materials

Evaluating the Ecotoxicological Effects of Microplastics on Terrestrial Passerines: Insights from Eurasian Tree Sparrows

Mo Li [1,3,4†], Jun Wang[1,4,†], Weiyue Meng[1,4], Liqiang Du[5], Dongming Li[3*], Yanfeng Sun[2,3*]

Affiliation:

[1] College of Life Sciences, Cangzhou Normal University, Cangzhou 061001, China.

[2] Hebei Key Laboratory of Bohai Rim Biomass Materials, Cangzhou Normal University, Cangzhou 061001, China

[3] Hebei Key Laboratory of Animal Physiology, Biochemistry and Molecular Biology, Hebei Collaborative Innovation Center for Eco-Environment, Ministry of Education Key Laboratory of Molecular and Cellular Biology, College of Life Sciences, Hebei Normal University, Shijiazhuang 050024, China.

[4] College of Marine Resources & Environment, Hebei Normal University of Science & Technology, Qinhuangdao 066600, China.

[5] Ocean College, Hebei Agricultural University, Qinhuangdao 066003, China.

[*] **Corresponding authors:**

Dongming Li (lidongming@hebtu.edu.cn); Yanfeng Sun (sunyanfeng@hebau.edu.cn).

Supplementary materials include 6 tables 5 figures and in Page S3-16:

Table S1. The *post hoc* multiple comparison for body mass, core temperature and food intake (***Page S3-5***)

Table S2. Reads per sample by 16S rRNA sequencing ***(Page S6-7)***

Table S3. The OTU counts of each sample ***(Page S8)***

Table S4. Relative abundance variations of the top 10 phyla ***(Page S9)***

Table S5. Pairwise PERMANOVA comparisons of gut microbial β-diversity ***(Page S10)***

Table S6. Differential bacterial taxa identified by Lefse analysis *(Page S11)*

Table S7. The relative frequency percentage of top 10 predicted function and pairwise

comparison results ***(Page S12)***

Figure S1. Scanning electron microscope images of PS-MPs ***(Page S13)***

Figure S2. Intestinal tissue section ***(Page S14)***

Figure S3. Rarefaction curve ***(Page S15)***

Figure S4. PERMANOVA analysis between different sexes ***(Page S16)***

Figure S5. Relative abundance of predicted function of microbial communities ***(Page S17)***

Table S1. The *post hoc* multiple comparison results for body mass, core temperature and food intake across all time points from day 1 to day 21. Pairwise comparisons were conducted by Bonferroni method, and *P*-values were corrected using BH method. Significant values were shown in bold.

Variable	time	1d	2d	3d	4d	5d	6d	7d	8d	9d	10d	11d	12d	13d	14d	15d	16d	17d	18d	19d	20d	21d
Body mass	1d		1.000	1.000	1.000	**0.006**	**<0.001**	**0.012**	**<0.001**	**<0.001**	**<0.001**	**<0.001**	**<0.001**	**<0.001**	**<0.001**	**<0.001**	**<0.001**	**<0.001**	**<0.001**	**<0.001**	**<0.001**	**<0.001**
	2d	1.000		1.000	0.853	**<0.001**	**<0.001**	**0.004**	**<0.001**	**<0.001**	**<0.001**	**<0.001**	**<0.001**	**<0.001**	**<0.001**	**<0.001**	**<0.001**	**<0.001**	**<0.001**	**<0.001**	**<0.001**	**<0.001**
	3d	1.000	1.000		1.000	**<0.001**	**<0.001**	**<0.001**	**<0.001**	**<0.001**	**<0.001**	**<0.001**	**<0.001**	**<0.001**	**<0.001**	**<0.001**	**<0.001**	**<0.001**	**<0.001**	**<0.001**	**<0.001**	**<0.001**
	4d	1.000	0.853	1.000		**0.011**	**<0.001**	**0.018**	**<0.001**	**<0.001**	**<0.001**	**<0.001**	**<0.001**	**<0.001**	**<0.001**	**<0.001**	**<0.001**	**<0.001**	**<0.001**	**<0.001**	**<0.001**	**<0.001**
	5d	**0.006**	**<0.001**	**<0.001**	**0.011**		**0.027**	1.000	**<0.001**	**0.007**	**<0.001**	**<0.001**	**<0.001**	**<0.001**	**<0.001**	**<0.001**	**<0.001**	**<0.001**	**<0.001**	**<0.001**	**<0.001**	**<0.001**
	6d	**<0.001**	**<0.001**	**<0.001**	**<0.001**	**0.027**		1.000	0.063	0.893	0.050	**0.010**	**0.005**	**0.004**	**0.004**	**0.009**	**0.006**	**<0.001**	**<0.001**	**<0.001**	**<0.001**	**<0.001**
	7d	**0.012**	**0.004**	**<0.001**	**0.018**	1.000	1.000		0.392	**0.030**	**0.019**	**<0.001**	**0.003**	**0.002**	**0.002**	**0.002**	**0.003**	**<0.001**	**<0.001**	**<0.001**	**<0.001**	**<0.001**
	8d	**<0.001**	**<0.001**	**<0.001**	**<0.001**	**<0.001**	0.063	0.392		1.000	1.000	1.000	0.213	0.061	0.089	0.207	0.055	**0.005**	**0.006**	**0.003**	**<0.001**	**<0.001**
	9d	**<0.001**	**<0.001**	**<0.001**	**<0.001**	**0.007**	0.893	**0.030**	1.000		1.000	0.384	0.553	0.128	0.062	0.087	0.069	**0.003**	**0.003**	**0.002**	**<0.001**	**<0.001**
	10d	**<0.001**	**<0.001**	**<0.001**	**<0.001**	**<0.001**	0.050	**0.019**	1.000	1.000		1.000	1.000	0.150	0.203	0.327	0.063	**0.003**	**0.003**	**0.001**	**<0.001**	**<0.001**
	11d	**<0.001**	**<0.001**	**<0.001**	**<0.001**	**<0.001**	**0.010**	**<0.001**	1.000	0.384	1.000		1.000	0.860	0.305	0.379	0.202	**0.002**	**0.002**	**0.002**	**<0.001**	**<0.001**
	12d	**<0.001**	**<0.001**	**<0.001**	**<0.001**	**<0.001**	**0.005**	**0.003**	0.213	0.553	1.000	1.000		1.000	1.000	1.000	1.000	**0.009**	**0.009**	**0.002**	**<0.001**	**<0.001**
	13d	**<0.001**	**<0.001**	**<0.001**	**<0.001**	**<0.001**	**0.004**	**0.002**	0.061	0.128	0.150	0.860	1.000		1.000	1.000	1.000	0.053	0.149	**0.007**	**<0.001**	**<0.001**
	14d	**<0.001**	**<0.001**	**<0.001**	**<0.001**	**<0.001**	**0.004**	**0.002**	0.089	0.062	0.203	0.305	1.000	1.000		1.000	1.000	**0.030**	**0.042**	**0.009**	**<0.001**	**<0.001**
	15d	**<0.001**	**<0.001**	**<0.001**	**<0.001**	**<0.001**	**0.009**	**0.002**	0.207	0.087	0.327	0.379	1.000	1.000	1.000		1.000	**0.002**	**0.003**	**0.004**	**<0.001**	**<0.001**
	16d	**<0.001**	**<0.001**	**<0.001**	**<0.001**	**<0.001**	**0.006**	**0.003**	0.055	0.069	0.063	0.202	1.000	1.000	1.000	1.000		0.334	1.000	0.131	**0.001**	**<0.001**
	17d	**<0.001**	**<0.001**	**<0.001**	**<0.001**	**<0.001**	**<0.001**	**<0.001**	**0.005**	**0.003**	**0.003**	**0.002**	**0.009**	0.053	**0.030**	**0.002**	0.334		1.000	1.000	0.067	**0.013**
	18d	**<0.001**	**<0.001**	**<0.001**	**<0.001**	**<0.001**	**<0.001**	**<0.001**	**0.006**	**0.003**	**0.003**	**0.002**	**0.009**	0.149	**0.042**	**0.003**	1.000	1.000		1.000	**0.009**	**0.001**
	19d	**<0.001**	**<0.001**	**<0.001**	**<0.001**	**<0.001**	**<0.001**	**<0.001**	**0.003**	**0.002**	**0.001**	**0.002**	**0.002**	**0.007**	**0.009**	**0.004**	0.131	1.000	1.000		0.442	**0.004**
	20d	**<0.001**	**<0.001**	**<0.001**	**<0.001**	**<0.001**	**<0.001**	**<0.001**	**<0.001**	**<0.001**	**<0.001**	**<0.001**	**<0.001**	**<0.001**	**<0.001**	**<0.001**	**0.001**	0.067	**0.009**	0.442		1.000
	21d	**<0.001**	**<0.001**	**<0.001**	**<0.001**	**<0.001**	**<0.001**	**<0.001**	**<0.001**	**<0.001**	**<0.001**	**<0.001**	**<0.001**	**<0.001**	**<0.001**	**<0.001**	**<0.001**	**0.013**	**0.001**	**0.004**	1.000	
Core temperature	1d		**0.019**	**0.029**	0.144	1.000	1.000	1.000	1.000	**<0.001**	**<0.001**	0.262	0.349	1.000	**0.030**	**0.038**	0.251	0.289	**<0.001**	**<0.001**	0.828	1.000
	2d	**0.019**		1.000	1.000	1.000	**0.033**	**0.008**	1.000	0.284	**0.021**	1.000	1.000	1.000	1.000	1.000	1.000	1.000	0.422	**0.002**	1.000	0.050

Variable	time	1d	2d	3d	4d	5d	6d	7d	8d	9d	10d	11d	12d	13d	14d	15d	16d	17d	18d	19d	20d	21d
	3d	**0.029**	1.000		1.000	1.000	**0.005**	**0.014**	1.000	1.000	0.173	1.000	1.000	1.000	1.000	1.000	1.000	1.000	1.000	**0.011**	1.000	**0.044**
	4d	0.144	1.000	1.000		1.000	**0.002**	**<0.001**	1.000	0.687	**0.017**	1.000	1.000	1.000	1.000	1.000	1.000	1.000	0.675	**0.002**	1.000	**0.004**
	5d	1.000	1.000	1.000	1.000		0.683	0.515	1.000	1.000	0.078	1.000	1.000	1.000	1.000	1.000	1.000	1.000	1.000	**0.006**	1.000	0.565
	6d	1.000	**0.033**	**0.005**	**0.002**	0.683		1.000	1.000	**<0.001**	**<0.001**	**0.034**	0.055	1.000	**<0.001**	**0.003**	**0.004**	**0.036**	**<0.001**	**<0.001**	0.121	1.000
	7d	1.000	**0.008**	**0.014**	**<0.001**	0.515	1.000		1.000	**<0.001**	**<0.001**	**0.009**	**0.028**	0.777	**0.002**	**0.002**	**0.002**	**0.019**	**<0.001**	**<0.001**	0.179	1.000
	8d	1.000	1.000	1.000	1.000	1.000	1.000	1.000		**0.007**	**<0.001**	0.578	1.000	1.000	**0.037**	0.295	0.988	1.000	**0.011**	**<0.001**	1.000	0.128
	9d	**<0.001**	0.284	1.000	0.687	1.000	**<0.001**	**<0.001**	**0.007**		1.000	1.000	1.000	0.065	1.000	1.000	1.000	1.000	1.000	0.069	1.000	**<0.001**
	10d	**<0.001**	**0.021**	0.173	**0.017**	0.078	**<0.001**	**<0.001**	**<0.001**	1.000		0.487	**0.018**	**<0.001**	0.503	0.199	0.066	**0.029**	1.000	0.537	1.000	**<0.001**
	11d	0.262	1.000	1.000	1.000	1.000	**0.034**	**0.009**	0.578	1.000	0.487		1.000	1.000	1.000	1.000	1.000	1.000	1.000	**0.015**	1.000	**<0.001**
	12d	0.349	1.000	1.000	1.000	1.000	0.055	**0.028**	1.000	1.000	**0.018**	1.000		1.000	1.000	1.000	1.000	1.000	1.000	**0.003**	1.000	**<0.001**
	13d	1.000	1.000	1.000	1.000	1.000	1.000	0.777	1.000	0.065	**<0.001**	1.000	1.000		0.161	0.705	1.000	1.000	**0.004**	**<0.001**	1.000	**0.008**
	14d	**0.030**	1.000	1.000	1.000	1.000	**<0.001**	**0.002**	**0.037**	1.000	0.503	1.000	1.000	0.161		1.000	1.000	1.000	1.000	**0.003**	1.000	**<0.001**
	15d	**0.038**	1.000	1.000	1.000	1.000	**0.003**	**0.002**	0.295	1.000	0.199	1.000	1.000	0.705	1.000		1.000	1.000	1.000	**0.002**	1.000	**<0.001**
	16d	0.251	1.000	1.000	1.000	1.000	**0.004**	**0.002**	0.988	1.000	0.066	1.000	1.000	1.000	1.000	1.000		1.000	1.000	**0.008**	1.000	**<0.001**
	17d	0.289	1.000	1.000	1.000	1.000	**0.036**	**0.019**	1.000	1.000	**0.029**	1.000	1.000	1.000	1.000	1.000	1.000		1.000	**0.001**	1.000	**<0.001**
	18d	**<0.001**	0.422	1.000	0.675	1.000	**<0.001**	**<0.001**	**0.011**	1.000	1.000	1.000	1.000	**0.004**	1.000	1.000	1.000	1.000		**0.004**	1.000	**<0.001**
	19d	**<0.001**	**0.002**	**0.011**	**0.002**	**0.006**	**<0.001**	**<0.001**	**<0.001**	0.069	0.537	**0.015**	**0.003**	**<0.001**	**0.003**	**0.002**	**0.008**	**0.001**	**0.004**		1.000	**<0.001**
	20d	0.828	1.000	1.000	1.000	1.000	0.121	0.179	1.000	1.000	1.000	1.000	1.000	1.000	1.000	1.000	1.000	1.000	1.000	1.000		**0.023**
	21d	1.000	0.050	**0.044**	**0.004**	0.565	1.000	1.000	0.128	**<0.001**	**<0.001**	**<0.001**	**<0.001**	**0.008**	**<0.001**	**<0.001**	**<0.001**	**<0.001**	**<0.001**	**<0.001**	**0.023**	
Food intake	1d		1.000	**<0.001**	**<0.001**	**<0.001**	**<0.001**	**<0.001**	**<0.001**	**0.017**	**<0.001**	**<0.001**	**<0.001**	**<0.001**	**<0.001**	**<0.001**	**<0.001**	**<0.001**	**<0.001**	**<0.001**	**<0.001**	**<0.001**
	2d	1.000		**<0.001**	**<0.001**	**0.002**	**0.002**	**0.002**	0.062	1.000	**<0.001**	0.121	0.080	**0.016**	**<0.001**	**<0.001**	**<0.001**	**<0.001**	**<0.001**	**<0.001**	**<0.001**	**0.015**
	3d	**<0.001**	**<0.001**		0.801	1.000	1.000	1.000	1.000	1.000	1.000	1.000	1.000	1.000	0.761	0.215	**0.014**	**0.004**	**<0.001**	0.155	**0.001**	1.000
	4d	**<0.001**	**<0.001**	0.801		1.000	1.000	1.000	1.000	**0.046**	1.000	1.000	1.000	1.000	1.000	1.000	1.000	1.000	0.079	1.000	0.298	1.000
	5d	**<0.001**	**0.002**	1.000	1.000		1.000	1.000	1.000	1.000	1.000	1.000	1.000	1.000	1.000	1.000	1.000	0.604	**0.022**	1.000	0.155	1.000
	6d	**<0.001**	**0.002**	1.000	1.000	1.000		1.000	1.000	1.000	1.000	1.000	1.000	1.000	1.000	1.000	0.494	0.173	**0.009**	0.853	**0.009**	1.000

Variable	time	1d	2d	3d	4d	5d	6d	7d	8d	9d	10d	11d	12d	13d	14d	15d	16d	17d	18d	19d	20d	21d
	7d	**<0.001**	**0.002**	1.000	1.000	1.000	1.000		1.000	1.000	1.000	1.000	1.000	1.000	0.579	**0.045**	**0.005**	**<0.001**	**<0.001**	**0.027**	**<0.001**	1.000
	8d	**<0.001**	0.062	1.000	1.000	1.000	1.000	1.000		0.753	1.000	1.000	1.000	1.000	0.262	**0.004**	**<0.001**	**<0.001**	**<0.001**	**0.014**	**<0.001**	1.000
	9d	**0.017**	1.000	1.000	**0.046**	1.000	1.000	1.000	0.753		0.617	0.244	0.846	0.233	**0.049**	**<0.001**	**<0.001**	**<0.001**	**<0.001**	**<0.001**	**<0.001**	0.104
	10d	**<0.001**	**<0.001**	1.000	1.000	1.000	1.000	1.000	1.000	0.617		1.000	1.000	1.000	1.000	1.000	0.179	0.061	**<0.001**	1.000	0.001	1.000
	11d	**<0.001**	0.121	1.000	1.000	1.000	1.000	1.000	1.000	0.244	1.000		1.000	1.000	1.000	0.244	**0.039**	**0.005**	**<0.001**	0.107	**0.002**	1.000
	12d	**<0.001**	0.080	1.000	1.000	1.000	1.000	1.000	1.000	0.846	1.000	1.000		1.000	1.000	0.255	**0.002**	**0.009**	**<0.001**	0.245	**0.002**	1.000
	13d	**<0.001**	**0.016**	1.000	1.000	1.000	1.000	1.000	1.000	0.233	1.000	1.000	1.000		1.000	**0.003**	**0.002**	**<0.001**	**<0.001**	0.056	**0.005**	1.000
	14d	**<0.001**	**<0.001**	0.761	1.000	1.000	1.000	0.579	0.262	**0.049**	1.000	1.000	1.000	1.000		1.000	1.000	0.573	0.001	1.000	0.235	1.000
	15d	**<0.001**	**<0.001**	0.215	1.000	1.000	1.000	**0.045**	**0.004**	**<0.001**	1.000	0.244	0.255	**0.003**	1.000		1.000	1.000	0.198	1.000	1.000	1.000
	16d	**<0.001**	**<0.001**	**0.014**	1.000	1.000	0.494	**0.005**	**<0.001**	**<0.001**	0.179	**0.039**	**0.002**	**0.002**	1.000	1.000		1.000	0.804	1.000	1.000	1.000
	17d	**<0.001**	**<0.001**	**0.004**	1.000	0.604	0.173	**<0.001**	**<0.001**	**<0.001**	0.061	**0.005**	**0.009**	**<0.001**	0.573	1.000	1.000		1.000	1.000	1.000	0.577
	18d	**<0.001**	**<0.001**	**<0.001**	0.079	**0.022**	**0.009**	**<0.001**	**<0.001**	**<0.001**	**<0.001**	**<0.001**	**<0.001**	**<0.001**	0.001	0.198	0.804	1.000		1.000	1.000	**0.002**
	19d	**<0.001**	**<0.001**	0.155	1.000	1.000	0.853	**0.027**	**0.014**	**<0.001**	1.000	0.107	0.245	0.056	1.000	1.000	1.000	1.000	1.000		1.000	0.406
	20d	**<0.001**	**<0.001**	**0.001**	0.298	0.155	**0.009**	**<0.001**	**<0.001**	**<0.001**	0.001	**0.002**	**0.002**	**0.005**	0.235	1.000	1.000	1.000	1.000	1.000		0.056
	21d	**<0.001**	**0.015**	1.000	1.000	1.000	1.000	1.000	1.000	0.104	1.000	1.000	1.000	1.000	1.000	1.000	1.000	0.577	**0.002**	0.406	0.056	

Table S2. Reads per sample by 16S rRNA sequencing. Abbreviations: C, control group; LL, large particle-low dosage group; LH, large particle-high dosage group; SL, small particle-low dosage group; SH, small particle-high dosage group.

Sample ID	Raw Reads	Clean Reads	Effective Reads	AvgLen(bp)	GC(%)	Q20(%)	Q30(%)	Effective(%)
C-1	160182	159843	156243	417	54.27	99.25	96.83	97.54
C-2	141451	141142	137706	422	51.76	99.26	96.78	97.35
C-3	159949	159603	155261	415	51.02	99.33	97	97.07
C-4	160142	159783	155660	423	50.15	99.23	96.61	97.2
C-5	159960	159581	157531	423	47.66	99.25	96.61	98.48
C-6	131112	130785	126873	418	53.47	99.27	96.84	96.77
C-7	157623	157250	154421	425	51.45	99.23	96.68	97.97
C-8	159971	159631	153203	419	55	99.28	96.93	95.77
C-9	140517	140154	137586	420	52.38	99.27	96.79	97.91
C-10	110072	109808	106186	418	54.43	99.31	97.01	96.47
LL-1	159992	159607	154478	418	56.13	99.28	96.94	96.55
LL-2	157871	157497	152078	417	53.87	99.27	96.88	96.33
LL-3	159962	159495	156110	424	54.77	99.3	96.9	97.59
LL-4	160245	159840	158107	428	46.74	99.25	96.52	98.67
LL-5	160060	159695	153010	417	54.35	99.29	96.93	95.6
LL-6	160151	159742	153297	418	54.5	99.27	96.89	95.72
LL-7	160471	160047	153204	418	54.51	99.29	96.93	95.47
LL-8	160188	159757	155567	419	53.7	99.26	96.82	97.12
LL-9	159586	159218	156192	423	52.55	99.28	96.83	97.87
LH-1	160062	159634	154539	418	54.28	99.26	96.83	96.55
LH-2	160145	159747	154097	419	54.63	99.27	96.83	96.22
LH-3	159921	159520	152570	418	54.05	99.27	96.88	95.4
LH-4	160080	159678	156430	420	53.37	99.21	96.67	97.72
LH-5	159821	159440	155501	425	55.34	99.29	96.87	97.3
LH-6	160019	159621	154362	418	54.49	99.26	96.84	96.46
LH-7	160147	159749	155071	421	50.36	99.2	96.58	96.83
LH-8	159826	159420	152022	418	54.83	99.25	96.79	95.12
LH-9	159959	159627	152931	415	51.74	99.28	96.85	95.61
SL-1	120796	120527	116705	417	53.72	99.28	96.9	96.61
SL-2	119588	119284	116760	424	52.81	99.32	96.98	97.64
SL-3	159994	159635	156052	411	51.53	99.36	97.11	97.54
SL-4	104505	104255	101153	416	53.86	99.3	96.97	96.79
SL-5	136578	136236	131433	417	54.44	99.27	96.91	96.23
SL-6	159976	159631	155138	420	53.63	99.28	96.87	96.98
SL-7	159967	159597	155487	421	55.53	99.3	96.96	97.2
SL-8	121935	121688	117140	419	53.94	99.29	96.93	96.07
SL-9	159713	159314	156015	425	51.47	99.28	96.8	97.68
SL-10	118692	118428	114617	416	54.34	99.28	96.94	96.57
SH-1	160000	159632	156922	425	48.32	99.22	96.51	98.08
SH-2	159802	159392	157203	425	46.8	99.22	96.48	98.37

Sample ID	Raw Reads	Clean Reads	Effective Reads	AvgLen(bp)	GC(%)	Q20(%)	Q30(%)	Effective(%)
SH-3	152452	152123	146562	422	53.84	99.27	96.86	96.14
SH-4	160326	159943	157140	426	53.15	99.28	96.82	98.01
SH-5	160146	159688	156141	421	53.06	99.23	96.67	97.5
SH-6	160322	159932	155822	415	54.34	99.26	96.87	97.19
SH-7	159944	159563	156085	422	52.43	99.28	96.84	97.59
SH-8	138171	137808	133015	418	54.52	99.28	96.91	96.27
SH-9	160295	159909	156830	424	51.56	99.29	96.84	97.84
SH-10	160108	159677	155844	421	53.84	99.28	96.85	97.34

Table S3. The OTU counts of each sample. Abbreviations: C, control group; LL, large particle-low dosage group; LH, large particle-high dosage group; SL, small particle-low dosage group; SH, small particle-high dosage group.

Sample ID	OTUNum	Sample ID	OTUNum	Sample ID	OTUNum	Sample ID	OTUNum
C-1	554	LL-3	942	LH-6	1012	SL-9	553
C-2	607	LL-4	807	LH-7	1047	SL-10	601
C-3	645	LL-5	1037	LH-8	965	SH-1	601
C-4	600	LL-6	942	LH-9	903	SH-2	568
C-5	649	LL-7	978	SL-1	625	SH-3	860
C-6	655	LL-8	984	SL-2	585	SH-4	648
C-7	622	LL-9	890	SL-3	616	SH-5	896
C-8	843	LH-1	1022	SL-4	630	SH-6	924
C-9	672	LH-2	945	SL-5	601	SH-7	918
C-10	609	LH-3	947	SL-6	604	SH-8	1000
LL-1	1059	LH-4	994	SL-7	633	SH-9	932
LL-2	1074	LH-5	961	SL-8	593	SH-10	924

Table S4. Relative abundance variations of the top 10 phyla across experimental groups. *Adjusted P* represent *P*-values adjusted by the BH method.

Phylum	*df*	*F/H*	*P*	*adjusted P*
Firmicutes	4	1.765	0.779	0.865
Proteobacteria	4	11.573	0.021	0.104
Cyanobacteria	4	1.251	0.870	0.870
Tenericutes	4	8.456	0.076	0.191
Bacteroidetes	4	12.217	0.016	0.104
Actinobacteria	4	5.594	0.232	0.331
Acidobacteria	4	6.477	0.166	0.310
Epsilonbacteraeota	4	9.724	0.045	0.151
Chloroflexi	4, 43	1.623	0.186	0.310
Verrucomicrobia	4, 43	1.062	0.387	0.484

Table S5. Pairwise PERMANOVA comparisons of gut microbial β-diversity among experimental groups. *P*-values were adjusted by the BH method, and significant values were shown in bold. Abbreviations: C, control group; LL, large particle-low dosage group; LH, large particle-high dosage group; SL, small particle-low dosage group; SH, small particle-high dosage group.

Group	C	LL	LH	SL	SH
C		**0.002**	**0.002**	**0.004**	**0.006**
LL	**0.002**		**0.002**	**0.002**	**0.003**
LH	**0.002**	**0.002**		**0.002**	**0.002**
SL	**0.004**	**0.002**	**0.002**		**0.003**
SH	**0.006**	**0.003**	**0.002**	**0.003**	

Table S6. Differential bacterial taxa identified by Lefse analysis (LDA > 3.0, *adjusted P* < 0.05). *Adjusted P* represent *P*-values adjusted by the BH method. Abbreviations: C, control group; LL, large particle-low dosage group; LH, large particle-high dosage group; SL, small particle-low dosage group; SH, small particle-high dosage group.

	bacterial taxa	enrich_group	LDA	*P*	*adjusted P*
1	k__Bacteria|p__Actinobacteria|c__Actinobacteria|o__Micromonosporales|f__Micromonosporaceae|g__uncultured_bacterium_f_Micromonosporaceae	C	3.159	0.037	0.045
2	k__Bacteria|p__Bacteroidetes|c__Bacteroidia|o__Bacteroidales|f__Marinilabiliaceae|g__uncultured_bacterium_f_Marinilabiliaceae	C	3.010	0.020	0.044
3	k__Bacteria|p__Bacteroidetes	LH	4.700	0.027	0.045
4	k__Bacteria|p__Bacteroidetes|c__Bacteroidia	LH	4.699	0.029	0.045
5	k__Bacteria|p__Bacteroidetes|c__Bacteroidia|o__Cytophagales|f__Amoebophilaceae|g__Candidatus_Cardinium	LH	4.372	0.001	0.007
6	k__Bacteria|p__Bacteroidetes|c__Bacteroidia|o__Cytophagales|f__Amoebophilaceae	LH	4.372	0.001	0.007
7	k__Bacteria|p__Bacteroidetes|c__Bacteroidia|o__Bacteroidales|f__Prevotellaceae	LH	3.914	0.014	0.039
8	k__Bacteria|p__Firmicutes|c__Bacilli|o__Lactobacillales|f__Enterococcaceae|g__Enterococcus	LH	3.384	0.001	0.008
9	k__Bacteria|p__Actinobacteria|c__Actinobacteria|o__Micrococcales|f__Micrococcaceae|g__Kocuria	LH	3.317	<0.001	0.007
10	k__Bacteria|p__Bacteroidetes|c__Bacteroidia|o__Chitinophagales	LH	3.308	0.039	0.045
11	k__Bacteria|p__Firmicutes|c__Clostridia|o__Clostridiales|f__Ruminococcaceae|g__uncultured_bacterium_f_Ruminococcaceae	LH	3.306	0.038	0.045
12	k__Bacteria|p__Bacteroidetes|c__Bacteroidia|o__Chitinophagales|f__Chitinophagaceae	LH	3.286	0.022	0.044
13	k__Bacteria|p__Firmicutes|c__Clostridia|o__Clostridiales|f__Ruminococcaceae|g__Subdoligranulum	LH	3.186	0.008	0.025
14	k__Bacteria|p__Bacteroidetes|c__Bacteroidia|o__Bacteroidales|f__Prevotellaceae|g__Prevotella	LH	3.029	0.015	0.039
15	k__Bacteria|p__Proteobacteria|c__Alphaproteobacteria|o__Rhizobiales|f__Xanthobacteraceae	LL	3.486	0.025	0.045
16	k__Bacteria|p__Proteobacteria|c__Alphaproteobacteria|o__Rhizobiales|f__Xanthobacteraceae|g__uncultured_bacterium_f_Xanthobacteraceae	LL	3.245	0.022	0.044
17	k__Bacteria|p__Proteobacteria|c__Alphaproteobacteria|o__Rhizobiales|f__Xanthobacteraceae|g__Bradyrhizobium	LL	3.195	0.029	0.045
18	k__Bacteria|p__Proteobacteria|c__Gammaproteobacteria|o__Steroidobacterales	LL	3.048	0.038	0.045
19	k__Bacteria|p__Proteobacteria|c__Gammaproteobacteria|o__Steroidobacterales|f__Steroidobacteraceae	LL	3.048	0.038	0.045
20	k__Bacteria|p__Proteobacteria|c__Gammaproteobacteria|o__Steroidobacterales|f__Steroidobacteraceae|g__uncultured_bacterium_f_Steroidobacteraceae	LL	3.021	0.032	0.045
21	k__Bacteria|p__Epsilonbacteraeota	SL	4.456	0.046	0.046
22	k__Bacteria|p__Epsilonbacteraeota|c__Campylobacteria	SL	4.456	0.046	0.046
23	k__Bacteria|p__Epsilonbacteraeota|c__Campylobacteria|o__Campylobacterales	SL	4.456	0.046	0.046
24	k__Bacteria|p__Spirochaetes	SL	3.429	0.045	0.046
25	k__Bacteria|p__Spirochaetes|c__Brachyspirae|o__Brachyspirales|f__Brachyspiraceae|g__Brachyspira	SL	3.412	0.003	0.009
26	k__Bacteria|p__Spirochaetes|c__Brachyspirae	SL	3.412	0.003	0.009
27	k__Bacteria|p__Spirochaetes|c__Brachyspirae|o__Brachyspirales	SL	3.412	0.003	0.009
28	k__Bacteria|p__Spirochaetes|c__Brachyspirae|o__Brachyspirales|f__Brachyspiraceae	SL	3.412	0.003	0.009

Table S7. The relative frequency percentage of top 10 predicted function and pairwise comparison results. Data were presented as Mean±SEM. *Adjusted P* represent *P*-values adjusted by the BH method. Abbreviations: C, control group; LL, large particle-low dosage group; LH, large particle-high dosage group; SL, small particle-low dosage group; SH, small particle-high dosage group.

Predicted function	C	LL	LH	SL	SH	C-LL		C-LH		C-SL		C-SH		BL-SL		BH-SH		BL-BH		SL-SH	
						P	*adjusted P*	*P*	*adjusted P*	*P*	*adjusted P*	*P*	*adjusted P*	*P*	*adjusted P*	*P*	*adjusted P*	*P*	*adjusted P*	*P*	*adjusted P*
Metabolic pathways	16.345±0.062	16.402±0.048	16.498±0.063	16.413±0.048	16.351±0.095	0.507	2.79	0.122	0.268	0.425	0.935	0.962	0.962	0.875	1.374	0.239	0.292	0.271	1.488	0.587	0.718
Biosynthesis of secondary metabolites	7.462±0.035	7.466±0.063	7.528±0.055	7.436±0.052	7.393±0.078	0.959	0.959	0.356	0.49	0.702	0.859	0.46	1.012	0.735	2.023	0.202	0.278	0.497	1.093	0.671	0.738
Biosynthesis of antibiotics	5.556±0.017	5.531±0.037	5.557±0.049	5.53±0.023	5.548±0.022	0.58	1.275	0.988	0.988	0.396	1.089	0.792	1.089	0.977	0.977	0.883	0.883	0.700	0.700	0.586	0.806
Microbial metabolism in diverse environments	4.328±0.059	4.453±0.08	4.509±0.063	4.434±0.049	4.31±0.04	0.253	2.781	0.065	0.719	0.209	2.303	0.814	0.995	0.847	1.553	0.025	0.091	0.617	0.754	0.079	0.433
Biosynthesis of amino acids	3.415±0.025	3.381±0.045	3.495±0.036	3.392±0.053	3.347±0.045	0.53	1.943	0.106	0.291	0.714	0.785	0.222	0.813	0.876	1.204	0.026	0.07	0.079	0.873	0.545	0.856
ABC transporters	3.3±0.061	3.261±0.078	3.153±0.135	3.185±0.102	3.328±0.119	0.717	0.877	0.368	0.45	0.377	1.383	0.846	0.93	0.584	2.142	0.37	0.406	0.523	0.959	0.399	0.732
Carbon metabolism	2.631±0.014	2.641±0.016	2.652±0.01	2.621±0.009	2.625±0.008	0.66	1.037	0.258	0.405	0.565	1.036	0.716	1.124	0.323	3.557	0.065	0.142	0.596	0.82	0.765	0.765
Ribosome	2.308±0.119	2.184±0.144	2.037±0.059	2.211±0.111	2.455±0.117	0.541	1.487	0.076	0.417	0.581	0.912	0.413	1.136	0.892	1.09	0.01	0.053	0.39	1.073	0.168	0.463
Purine metabolism	2.106±0.085	2.046±0.096	1.922±0.032	2.088±0.102	2.288±0.099	0.664	0.914	0.079	0.291	0.899	0.899	0.203	1.117	0.781	1.719	0.007	0.074	0.275	1.009	0.2	0.44
Two-component system	1.999±0.066	2.033±0.095	2.104±0.052	2.095±0.024	1.945±0.063	0.788	0.867	0.254	0.466	0.223	1.225	0.582	1.067	0.564	3.101	0.083	0.152	0.545	0.856	0.058	0.633

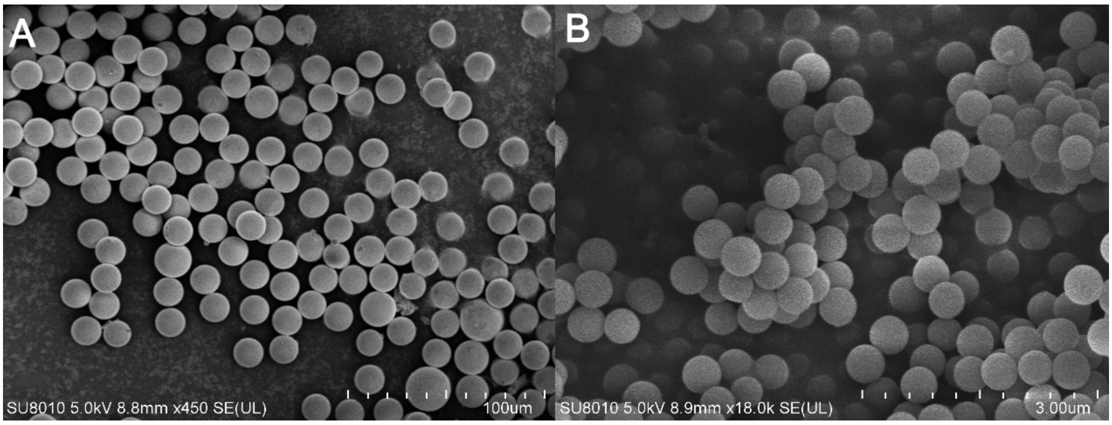

Figure S1. Scanning electron microscope images of polystyrene microplastics (PS-MPs) used in the present study (A: about 15μm in diameter; B: about 0.5 μm in diameter)

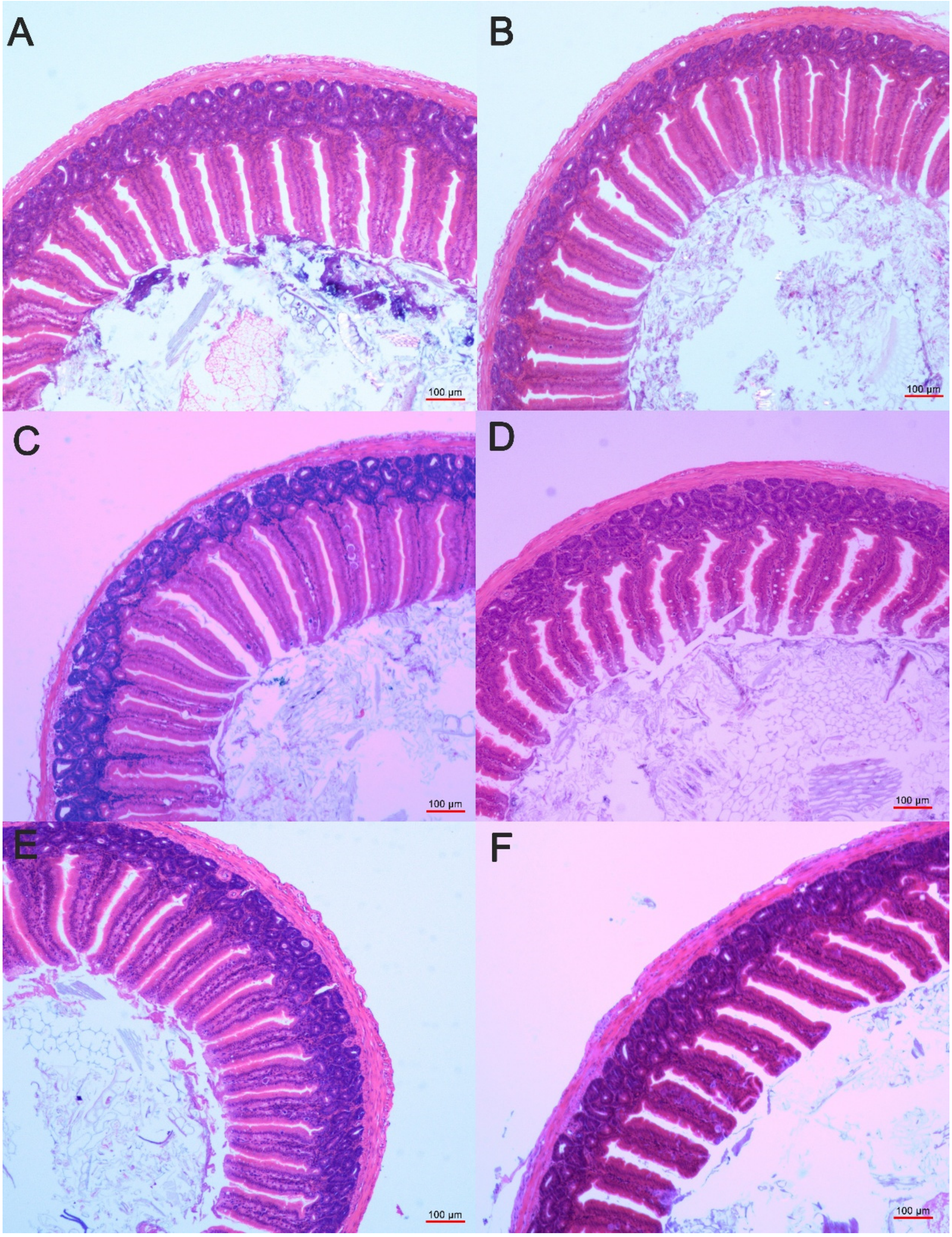

Figure S2. Intestinal tissue section of the Eurasian tree sparrows among different experimental groups (A, B: control group; C: large particle-low dosage group; D: small particle-low dosage group; E: large particle-high dosage group; F: small particle-high dosage group)

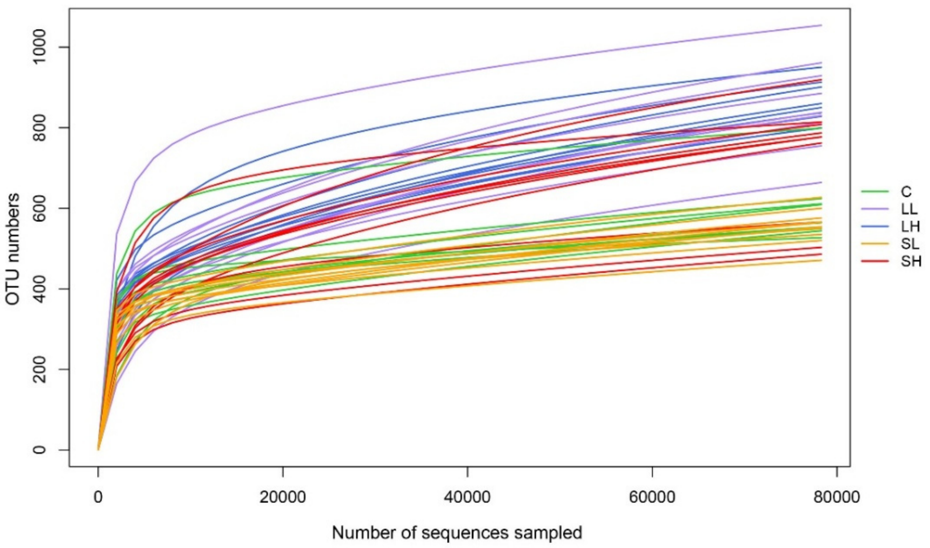

Figure S3. Rarefaction curve for all analyzed samples. Abbreviations: C, control group; LL, large particle-low dosage group; LH, large particle-high dosage group; SL, small particle-low dosage group; SH, small particle-high dosage group.

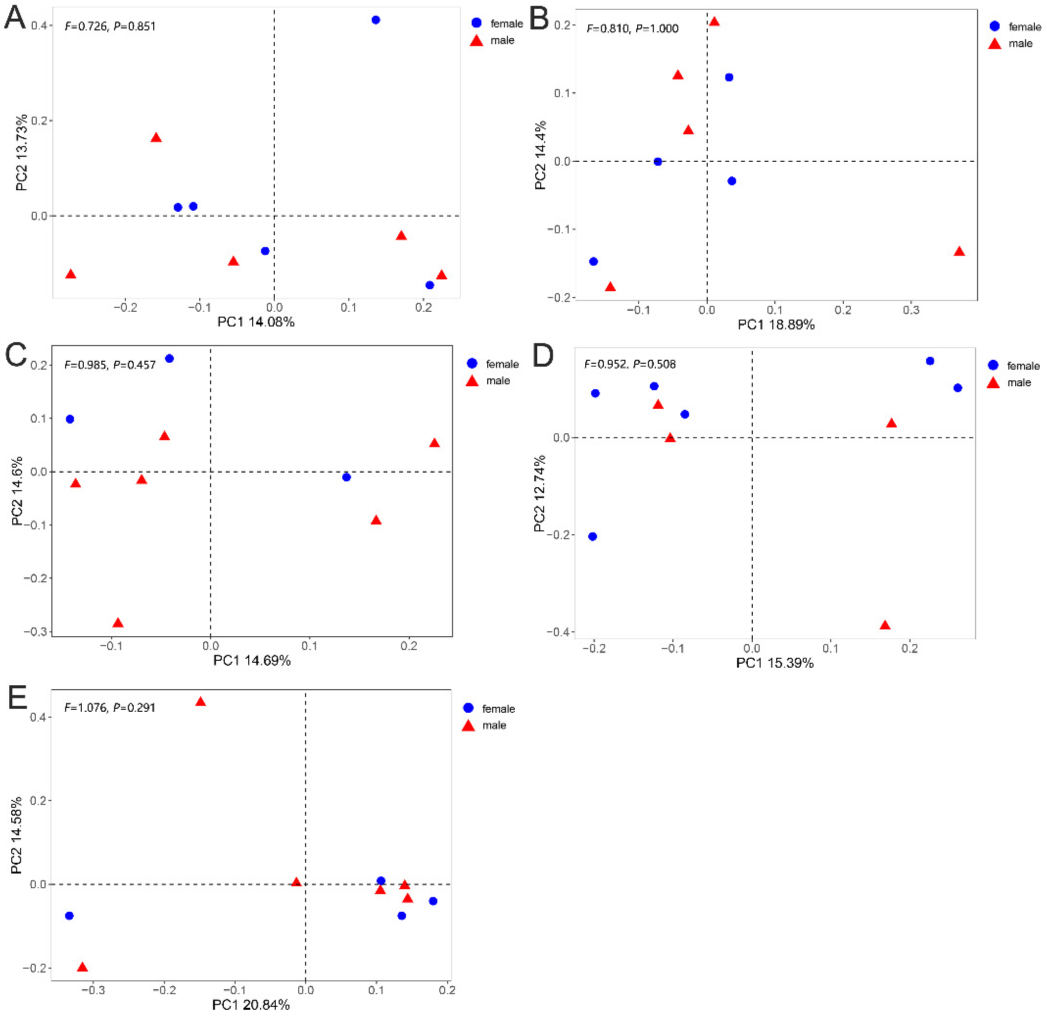

Figure S4. PERMANOVA analysis between different sexes across various groups. (A: control group; B: large particle-low dosage group; C: large particle-high dosage group; D: small particle-low dosage group; E: small particle-high dosage group).

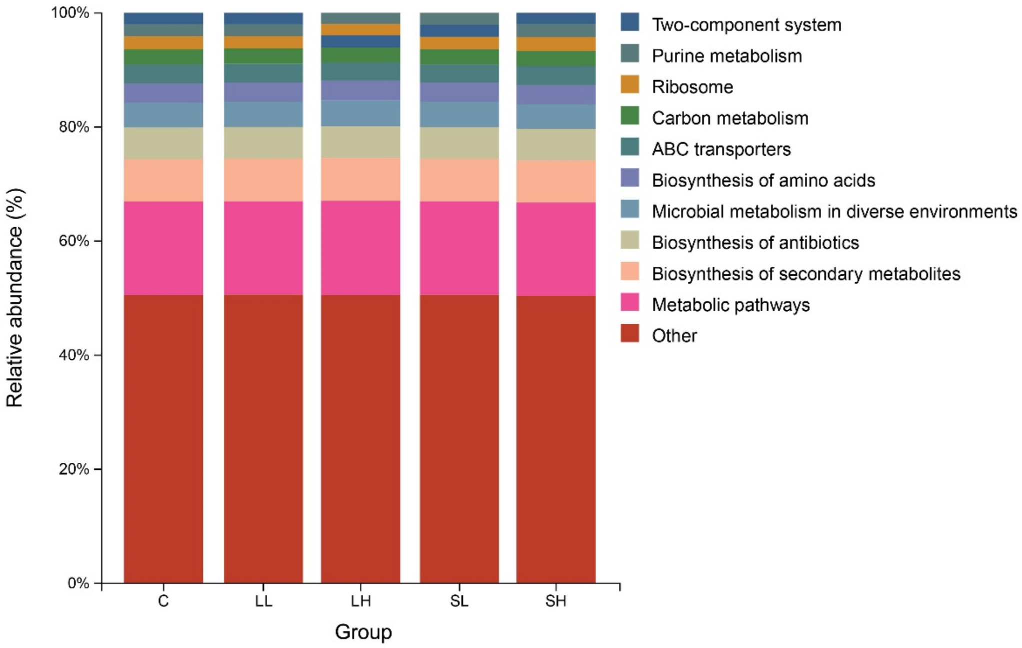

Figure S5. Relative abundance of predicted function of microbial communities across experimental groups. Abbreviations: C, control group; LL, large particle-low dosage group; LH, large particle-high dosage group; SL, small particle-low dosage group; SH, small particle-high dosage group.
